# Current state of esophageal cancer surgery in China: a national database analysis

**DOI:** 10.1186/s12885-019-6191-2

**Published:** 2019-11-08

**Authors:** Ming-Lian Qiu, Jian-Bo Lin, Xu Li, Rong-Gang Luo, Bo Liu, Jing-Wei Lin

**Affiliations:** 1 Thoracic Surgery Department, First Affiliated Hospital, Fujian Medical University, Fuzhou City, 350005 China; 2 Department of Medical Record Information, First Affiliated Hospital, Fujian Medical University, Fuzhou City, 350005 China; 3 Department of Health, Government of Fujian province, Fuzhou City, 350003 China

**Keywords:** Esophageal cancer, Surgery, China, Database

## Abstract

**Background:**

The present standard of surgical treatment for esophageal cancer is country dependent. The aim of the present study was to investigate the basic aspects of surgical procedures performed for esophageal cancer, and provide information about the present state of esophageal cancer surgery in China.

**Methods:**

Data were obtained from a database administered by the Chinese Ministry for Health. A total of 542 participating hospitals were divided into seven geographic areas, and 10% of hospitals in each area were randomly chosen for inclusion. All patients with esophageal cancer, who underwent esophagectomy in these participating hospitals from January 1 to December 31, 2015, were included in the present study. The clinical characteristics, stage of tumor at diagnosis, operation summary and outcomes, and histological findings of patients were extracted and analyzed.

**Results:**

The present study included 11,791 patients, and the average number of patients per hospital was 218. Squamous cell carcinoma was the most common pathological type, while the mid-esophagus was the most common location. Open procedures were performed in 63.8% of patients, while minimally invasive esophagectomy was performed in 36.2% of patients. Multiple approaches to transthoracic esophagectomy were utilized. Two-field lymphadenectomy was the most frequently performed (64.8%), followed by three-field lymphadenectomy (21.8%). Gastric tubes, thoracic duct ligation and postoperative enteral nutrition were implemented to minimize complications.

**Conclusion:**

The standard operative procedure and detailed technique for esophageal carcinoma surgery is presently being debated in China. This survey provides some basic information about the present state of esophageal cancer surgery countrywide.

## Background

Esophageal cancer (EC) is one of the most aggressive types of cancer, in which merely 15–25% of patients survive at five years after diagnosis [1]. The incidence of EC greatly varies by geographic location, with approximately 80% of cases occurring in developing countries. There is a high prevalence of EC in East Asia, eastern and southern Africa, and southern Europe [2, 3]. In China, EC is the fourth most common malignancy and fourth most common cause of malignancy-related death, with a reported prevalence of 52.1/100,000 in men and 24.4/100,000 in women [4]. It has been estimated that approximately 165,000 new cases of EC occur annually, and that approximately half of all EC surgeries worldwide are performed in China [5].

Surgery that comprises of radical resection of the esophagus and regional lymph nodes has been widely used for controlling EC in patients with locoregional disease. Since EC is often accompanied by the extensive involvement of cervical, thoracic and abdominal lymph nodes, and the esophagus is located deep in the posterior midline of the mediastinum, esophagectomy is a complex procedure with a high incidence of complications [6]. There is presently no standard surgical procedure, approach, extent of lymphadenectomy, or reconstructive technique, and the modalities of EC surgery are country dependent [7]. In China, these elements of management widely vary, and the surgeon characteristically attempts to balance surgical aggressiveness and safety when selecting a procedure.

Although large numbers of esophagectomies are performed in China, there is little information on the present state of EC surgery [8]. The aim of the present study was to investigate the basic aspects of surgical procedures performed for EC in China, and provide information to assist the Chinese Society for Esophageal Cancer to prepare the third edition of Clinical Practice Guidelines for the Diagnosis and Treatment of Esophageal Cancer in China by comparing the present finding with international guidelines.

## Methods

**Fig. 1:**
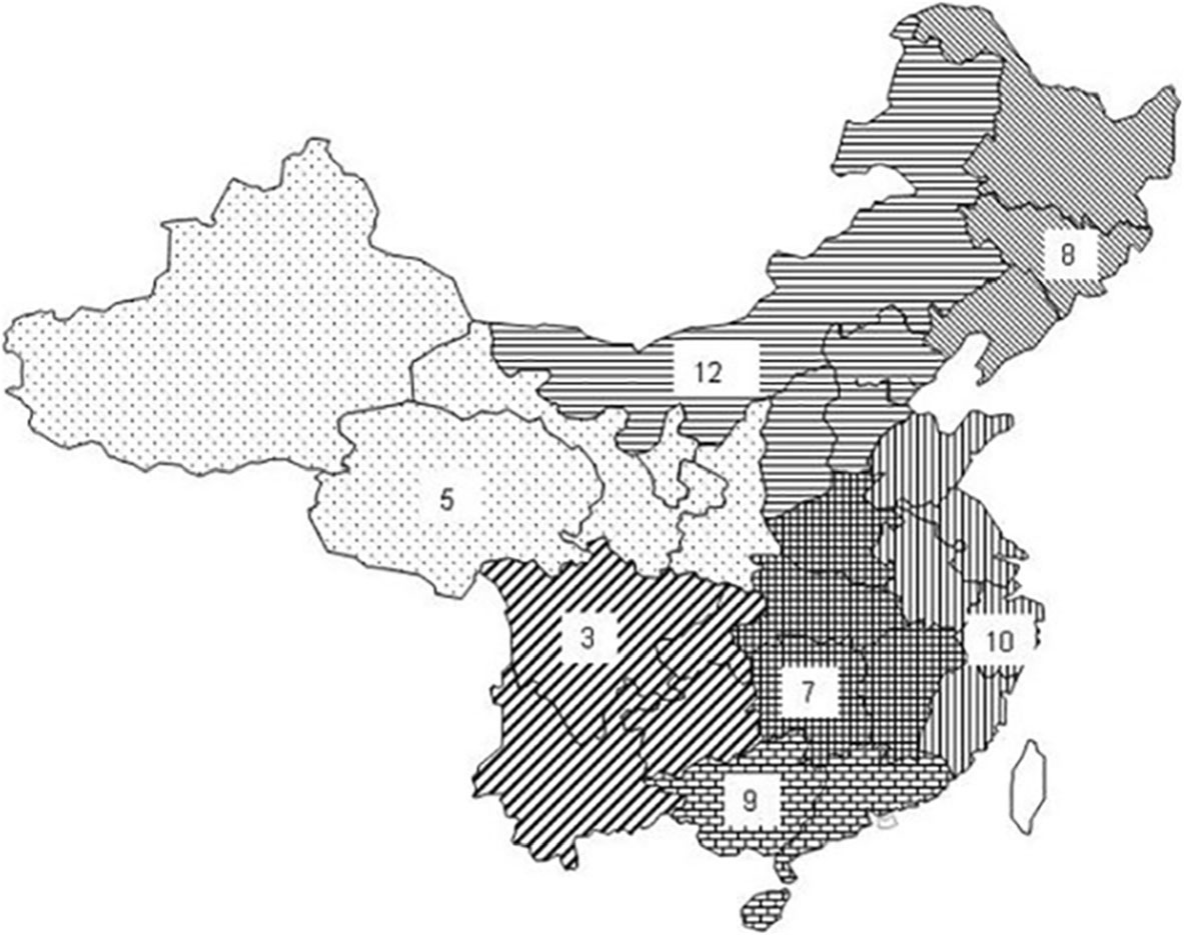
Geographic locations of the participating hospitals. (The picture is original, no conflict of copyright)

Data were obtained from a database administered by the Chinese Ministry for Health, which collects summaries of the diagnoses, management and outcomes of patients from 542 hospitals in China. The investigators were granted permission by the Health Department of Fujian Province Government to access the database. The hospitals were divided into seven geographic areas, and 10% of hospitals in each area were randomly chosen for inclusion (Fig. 1).

The inclusion criteria were the diagnosis of EC and esophagectomy from January 1, 2015 to December 31, 2015. Patients with esophageal-gastric junction cancer were excluded, because the Siewert classification is not routinely applied in China.

The collected data included the demographic patient characteristics, stage of the tumor at diagnosis, operation summary, outcomes, and histological findings. Twenty postgraduate students were trained to extract these data from the database. The data collection was approved by the Ethics Committee of Fujian Medical University (No. 2014078).

### Statistical analysis

All data were analyzed using a Microsoft Excel database, into which the working group entered data using a multiple-column format. All data were presented as absolute numbers and/or percentages. Differences in the incidence of anastomotic leakage and chylothorax were assessed using the Chi-square test for categorical variables. The analysis was performed using the SPSS software (version 12.0; SPSS, Chicago, IL, USA).

## Results

### Patient characteristics

Fifty-four hospitals or medical centers were randomly chosen from seven geographic areas of mainland China (Fig. 1). The median number of beds per hospital was 2100 (range: 1500–3750) and the median number of general thoracic surgery beds was 60 (range: 45–100) (Table 1). In 2015, a total of 11,791 esophagectomies were performed in these hospitals, and the average number performed by one department was 218. Squamous cell carcinoma was the most common pathological type, which comprised of 94.1% of all lesions, followed by adenocarcinoma (4.8%). The mid-esophagus was the most common location, and the percentages of tumors located in the upper, middle and lower third were 13.9, 59.8 and 26.3%, respectively. The most resectable lesions were at the late stage at diagnosis, in which 31.8% of patients were at stage II and 50.3% of patients were at stage III. Neoadjuvant therapy was infrequently administered, which was only given to 18.5% of patients. The relevant patient and tumor characteristics are listed in Table 2.

**Table 1:** Hospital locations and patient volumes

Area	No. of hospital	Beds of hospital range (median)	Beds of Thoracic Surgery Department range (median)	Case of surgery (average)
North-East	8	1800–2550 (2100)	45–75 (55)	1408 (176)
South-East	10	1850–2350 (2000)	45–80 (60)	2350 (235)
South	9	1800–2450 (2200)	50–75 (55)	1917 (213)
South-West	3	1950–3500 (2250)	50–95 (60)	518 (173)
Center	7	2200–3750 (2450)	50–100 (65)	2736 (390)
North-West	5	1500–2150 (1850)	50–75 (60)	503 (100)
North	12	1650–2500 (1950)	50–80 (55)	2359 (197)
Total	54	1500–3750 (2100)	45–100 (60)	11,791 (218)

**Table 2:** Demographic data and tumor characteristics (*N* = 11,791)

Variables	Number (%)
Age (year, median)	66.5 ± 3.2
Sex (M:F)	6836:4955
Neoadjuvant therapy	
Chemotherapy + radiology	728 (6.2)
Chemotherapy	1062 (9.0)
Radiotherapy	393 (3.3)
Adjuvant chemotherapy	2499 (21.2)
Location of the tumor	
Upper	1638 (13.9)
Middle	7047 (59.8)
Lower	3106 (26.3)
Oncological stage (pTNM)	
Stage I	2109 (17.9)
Stage II	3750 (31.8)
Stage III	5932 (50.3)
Margins	
R0	10,694 (90.7)
R1	696 (5.9)
R2	401 (3.4)
Pathological characteristic	
Squamous cell carcinoma	11,096 (94.1)
Adenocarcinoma	563 (4.8)
other	132 (1.1)

### Surgical approach

Open procedures were performed in 63.8% of patients, while minimally invasive esophagectomy (MIE) was performed in 36.2% of patients (Table 3). Among these open procedures, 97.4% were transthoracic, while 2.6% were transhiatal. Furthermore, the approaches to transthoracic esophagectomy were extremely diverse (Table 4). With regard to MIE, the McKeown approach (65.2%) was preferred by surgeons, followed by three-field lymph node dissection (LND) (23.2%) and the Ivor–Lewis approach (11.6%) (Table 5).

**Table 3:** Open versus MIE surgery (*N* = 11,791)

Issue	No. patient (%)
Open	7522 (63.8)
MIE	4180 (36.2)
Thoracoscopy+laparoscopy	3219
Thoracoscopy+laparotomy	865
Thoracotomy+laparoscopy	96
Not classified	89

**Table 4:** Approaches utilized in open surgery (*N* = 7522)

Issue	No. of incision	No. of patient (%)
Left Thoracotomy		
Left Thoracotomy	1	1215 (16.2)
Left Thoracotomy+cervical	2	173 (2.3)
Right Thoracotomy		
Ivor-Lewis	2	1043 (13.9)
Modified Ivor-Lewis	2	894 (11.8)
Mckeown	3	1231 (16.4)
Nathan	3	1170 (15.5)
3FLND	3	1599 (21.3)
Transhiatal	2	197 (2.6)

**Table 5:** Approaches used in MIE (*N* = 4180)

Issue	No. of incision	No. of patient (%)
Ivor-Lewis	2	485 (11.6)
Mckeown	3	2725 (65.2)
3 FLND	3	970 (23.2)

### Lymphadenectomy

Two-field LND was the most frequently performed (64.8%), while three-field LND was performed in 21.8% of patients. Furthermore, lower mediastinal and upper abdominal LND were performed in 13.4% of patients. The average number of lymph nodes harvested was 17.3, 21.6 and 7.2, respectively (Table 6).

**Table 6:** Extent of lymph node dissection (*N* = 11,791)

Issue	No. patient	LN harvested (average)
Lower mediastinum and upper abdominal dissection	1585 (13.4)	7.2
Two field dissection	7637 (64.8)	17.3
3 FLD	2569 (21.8)	21.6

### Anastomotic techniques and incidence of leakage

A stapling technique for intrathoracic anastomosis was favored, followed by hand-sewing (28.6% vs. 4.5%, Table 7). The incidence of intrathoracic leakage was 4.6% (4.6% stapling vs. 4.9% hand-sewing; X^2^ = 0.1, *P* > 0.05). Stapling and hand-sewing were utilized almost equally for cervical anastomoses (31.8% vs. 38.1%). The incidence of cervical leakage was 5.2% (6.4% stapling vs. 4.1% hand-sewing; X^2^ = 19.138, *P* < 0.001).

**Table 7:** Anastomotic techniques and incidence of leakage (*N* = 11,791)

Issue	No. of patient (%)	anastomotic leakage (%)
Intrathoracic	3899	181 (4.6)
Instrumental	3371 (28.6)	155 (4.6)
hand sewing	528 (4.5)	26 (4.9)
Cervical	7892	410 (5.2)
Instrumental	3746 (31.8)	240 (6.4)
hand sewing	4146 (35.1)	170 (4.1)

### Other elements of esophagectomy

Gastric tubes were used for the reconstruction in 63.8% of cases, while whole stomach reconstruction was performed in 34.4% of cases, and the colon or jejunum were seldom used (1.8%, Table 8). The thoracic duct was routinely resected or ligated in 52.9% of patients, while this was not routinely resected or ligated in the remaining 47.1% of patients. Jejunostomies (26.8%) or naso-jejunal feeding tubes (68.8%) were used for postoperative enteral nutrition, but merely 5% of patients did not receive enteral nutrition. Pyloroplasty was rarely performed during esophagectomy (1.2% of patients). The complications of esophagectomy are listed in Table 9. The mean hospital stay of all patients was 13.6 days.

**Table 8:** Other technical elements of esophageal cancer surgery (*N* = 11,791)

Issue	No. of patient (%)
Type of reconstruction	
Gastric tube	7527 (63.8)
Whole stomach	4051 (34.4)
Others (jejunum, colon)	213 (1.8)
Thoracic duct ligation	
Yes	6239 (52.9)
No	5522 (47.1)
Enteral nutrition	
jejunostomy	3145 (26.7)
Naso-jejunum feeding tube	8059 (68.3)
None	587 (5.0)
Pyloroplasty	
Yes	138 (1.2)
No	11,653 (98.8)

**Table 9:** Postoperative complications (*N* = 11,791)

Issue	No. (%)
Pneumonia	2736 (23.2)
Anastomosis leakage	660 (4.9)
Bleeding (need reoperation)	212 (1.7)
Respiratory failure (need mechanical ventilation)	366 (3.1)
Hoarseness	402 (3.4)
Chylothorax^a^	
Thoracic duct ligation (−)	66 (1.2)
Thoracic duct ligation (+)	13 (0.2)
Gastric empty delay	94 (0.8)
Re-admission (within 7 days)	155 (1.3)
In-hospital mortality	201 (1.7)

^a^The incidence of chylothorax was significant different between two groups, χ^2^ = 45.591, *p* < 0.001

## Discussion

Surgery for EC comprises of the removal of the primary lesion, LND and the restoration of the digestive tract. Such surgery is considered as one of the most extensive and traumatic of oncological surgical procedures, which not only involves a long operation time, but also a significant risk of morbidity [9].

In China, the optimal surgical procedure for EC remains an issue of debate, and the key controversial aspect is the extent of LND, in which there is presently no consensus. Published reports on this topic remain contradictory, and the choice of surgical approach is primarily driven by personal opinions and institutional preferences [10]. In general, there are two schools of thought that concern lymphadenectomy. According to the first school of thought, EC is often accompanied by extensive metastases to cervical, thoracic and abdominal lymph nodes, justifying the three-field lymphadenectomy. This enables for a more accurate pathological staging, and achieves better local control of the disease and long-term survival. This procedure was pioneered in Japan. However, at present, after approximately 30 years of its wide application, there is increasing evidence that extensive lymphadenectomy is associated with improved survival [11]. In the present cohort, 23.2% of patients underwent three-field LND in 2015.

In contrast, the other school of thought claims that extensive nodal dissection results in stage migration without improving the overall prognosis, and that associated complications can adversely affect postoperative recovery and long-term quality of life. This school attaches greater importance to safety and adjuvant therapy, when compared to lymphadenectomy, in the consideration that EC is at an advanced stage in most patients at the time of diagnosis, and that lymph node metastasis indicates the presence of systemic disease [12]. In the present cohort, two-field LND was performed in 64.8% of all cases, and an even more limited dissection was performed in 13.4% of cases.

The extent of LND is determined by the operative approach. The average number of lymph nodes harvested was 21.6, 17.3 and 7.2, respectively, for three-field, two-field, and lower mediastinal and upper abdominal LND. Left thoracotomy was once widely performed in China, because it is quicker and simpler than the right-sided two- or three-stage approach. The main advantages of left thoracotomy are that it permits for the exploration of the tumor, the dissection of the lesion, and the mobilization of the stomach through a single incision. This approach is contraindicated when the tumor is located at or cephalad to the aortic arch. In the present cohort, left thoracotomy was frequently performed, and employed in approximately 23% of open procedures.

A combined right thoracic and abdominal approach, which allows standard two-field LND, is presently the main favored procedure in EC surgery [13]. This procedure usually commences with an abdominal approach, which enables for the assessment of lymph node involvement, and the performance of gastrolysis, LND, jejunostomy, and sometimes, pyloroplasty. After the abdominal phase, right thoracotomy is performed, and intrathoracic lymphadenectomy and esophageal dissection is achieved. In the present study, the right thoracotomy approach was used in 45% of patients who underwent open surgery.

The McKeown procedure also allows for a standard two-field LND and a small component of the required neck LND [14]. An additional neck incision can enable for the transfer of the anastomosis from an intrathoracic to a cervical location. Anastomotic leakage is easier to manage in the cervical region. Approximately 21% of open procedures in the present cohort used the McKeown style, while three-field LND was chosen for 21% of open procedures. In addition, 2% of patients underwent esophagectomy via the transhiatal approach.

In the past decade, minimally invasive approaches have gained rapid acceptance, and have become an alternative means of performing EC surgery in China. By minimizing the size of incisions and reducing external surgical stress, MIE has become associated with significant perioperative advantages, including lower overall incidences of in-hospital pulmonary infections and shorter duration of stay in the intensive care unit [15]. MIE procedures limit the extent of possible traumatic stress, and thereby allow thoracic surgeons to achieve a good balance between oncological targets and safety [16]. In the present cohort, the ratio of MIE to open procedures was 30:70%. It was considered that when the percentage of early-stage lesions increases in the future, this ratio would also increase.

After the optimal surgical procedure and extent of LND for EC, the second major issue concerning esophagectomy is the minimization of complications [17]. Several techniques for reducing morbidity have been implemented. Anastomotic leakage has become a major concern, and the overall incidence in the present study was 5.6%. The anastomosis between the conduit and remaining esophagus can be located in the neck or chest. Several randomized trials have shown that both sites are equally safe, and have comparable morbidity [18–20]. A meta-analysis has shown no difference between these sites in the incidence of anastomotic leakage or stenosis [21]. In the present cohort, cervical anastomosis was preferred to intrathoracic anastomosis (66.9% vs. 32.1%), which was probably because leakage in the neck results in less morbidity, and is easier to manage.

Early enteral nutrition aims to accelerate the recovery from esophagectomy. Naso-jejunal feeding tubes are the most commonly used, because these are time-saving and less invasive, when compared to the other routes. These were employed in 68.8% of patients in the present study. Jejunostomy, which is also a good choice for prolonged enteral nutrition, was performed in 26.8% of patients in the present cohort.

The stomach is the most common conduit for restoration of the digestive tract during esophagectomy. In the present study, gastric tubes were the first choice for reconstruction, and this was used in 68.3% of all procedures, while the whole stomach was used in approximately one-third of patients. The advantages of the whole-stomach technique are that it is economical and time-saving. However, it has an obvious disadvantage of having a higher proportion of atelectasis.

There was a prominent discrepancy between the present study and published literature concerning the routine ligation of the thoracic duct during esophagectomy. Although the ligation of the thoracic duct has been shown to reduce the incidence of postoperative chyle leakage [22], this procedure was not performed in approximately half of patients in the present study, leading to a 1.2% incidence of chylothorax.

Pyloroplasty is rarely performed, because it is time-consuming. Even though the incidence of delayed gastric emptying is nearly 1%, most surgeons consider pyloroplasty to be unnecessary, and that gastric emptying improves after the administration of adequate enteral nutrition.

At present, a multidisciplinary treatment that comprises of surgery, chemotherapy and radiotherapy has been widely used, with a demonstrated improvement in prognosis. Two pivot studies revealed a significant overall survival benefit in neoadjuvant treatment [23, 24]. These concepts are slowly being accepted by Chinese surgeons. In the present survey, merely 18.5% of patients received neoadjuvant therapy, while 21% of patients received adjuvant therapy. Considering that 82.1% of patients were at stage II/III, more clinical trials are needed to help Chinese surgeons devise a more precise treatment strategy.

## Conclusion

To our knowledge, this is the first survey of EC surgery in China, which is a country that performs a huge number of EC operations annually. Unlike in other East Asian countries, such as Japan, in China, the standard operation and technique for EC surgery remains under debate. This survey provides some basic information about the present state of EC surgery in China. However, the data is limited, because merely the summarized information was available from the database, while the survival data was not available. Nonetheless, these preliminary findings may suggest directions for further studies. The present study could also assist the Chinese Society for Esophageal Cancer to prepare the third edition of the Clinical Practice Guidelines for the Diagnosis and Treatment of Esophageal Carcinoma by comparing the present finding with international guidelines.

## Data Availability

Not applicable.

## Abbreviations

EC: Esophageal cancer
LND: lymph node dissection
MIE: minimally invasive esophagectomy
